# The potential role of nitrous oxide in the etiology of autism spectrum disorder

**DOI:** 10.1038/tp.2016.89

**Published:** 2016-05-17

**Authors:** R E Frye, J Slattery

**Affiliations:** 1 Department of Pediatrics, University of Arkansas for Medical Sciences, Little Rock, AR, USA; 2 Division of Autism Research, Arkansas Children's Research Institute, Little Rock, AR, USA

Autism spectrum disorder (ASD) is a prevalent neurodevelopmental disorder that appears to have shared genetic and environmental etiologies.^1^ As the genetic causes have undergone intense investigation, the investigation of specific environmental agents that increase the risk of developing ASD has received less emphasis^2^ despite the fact that there is growing evidence for environmental factors such as toxicants^3^ and the enteric microbiome,^4, 5, 6^ just to name a few. Dr Fluegge, in his letter,^7^ outlines an interesting environmental influence, which may result in metabolic and behavioral abnormalities associated with ASD.

Nitrous oxide (N_2_O) is a greenhouse gas which originates, in part, from agriculture, fossil fuel combustion and other industrial sources, which has about 300 times the impact of CO_2_. Although N_2_O is a commonly used anesthetic in pediatrics, animal studies have pointed to adverse effects on the developing brain.^8^ Other studies have implicated N_2_O as a genotoxin,^9^ although this effect has been suggested to be indirect,^10^ perhaps through increases in oxidative stress.^11^ The animal studies have linked N_2_O with abnormalities in the maintenance of mitochondrial quality^12^ and mitochondrial function^13^ leading to abnormalities in synaptic dynamics in the developing brain.^13^ However, other epidemiological studies have failed to link it to adverse birth outcomes.^14^

An important caveat when considering individuals with ASD is that, for many, their metabolic systems appear to be under stress as many demonstrate abnormal redox^15^ and mitochondrial metabolism.^16^ In addition, mothers of children with ASD manifest some of these same metabolic abnormalities as their children.^17, 18^ Indeed, individuals with ASD may be particularly vulnerable to environmental perturbations which affect metabolic systems both prenatally and postnatally. Thus, the role of environmental agents such as N_2_O may be particularly significant in children with ASD, especially if other underlying conditions exist.

Particularly interesting in the letter from Dr Fluegge is the connection between N_2_O and the nicotinic alpha 7 cholinergic receptor. Indeed, abnormalities in regulation of the nicotinic alpha 7 cholinergic receptor can lead to autonomic dysfunction and inflammation, both of which are highly associated with ASD.^19, 20^ In fact, many lines of research have suggested decreased parasympathetic and increased sympathetic drive in many children with ASD, which could explain behavioral features of anxiety and irritability as well as physical symptoms such as chronic constipation.

Clearly, it is possible that children with ASD could be more sensitive to the effect of N_2_O, and BH_4_ could mitigate some of these effects by improving redox and nitric oxide metabolism.^21^ However, at this time, direct empirical evidence is lacking for this theory. Epidemiological studies examining the effect of environmental factors on the risk of developing of ASD have not examined environmental N_2_O nor has N_2_O exposure been integrated into an animal model of ASD. Thus, we must await further empirical study to provide a signal as to whether N_2_O has a significant role in the development or morbidity of ASD.
